# Promoting school readiness in children with developmental disabilities in LMICs

**DOI:** 10.3389/fpubh.2023.993642

**Published:** 2023-02-22

**Authors:** M. K. C. Nair, Rekha Radhakrishnan, Bolajoko O. Olusanya

**Affiliations:** ^1^NIMS-Spectrum Child Development Research Centre, Thiruvananthapuram, Kerala, India; ^2^Honorary Preschool Consultant, NIMS-Spectrum Child Development Research Centre, Thiruvananthapuram, Kerala, India; ^3^Centre for Healthy Start Initiative, Lagos, Nigeria

**Keywords:** school readiness, inclusive education, developmental disabilities, early detection, early intervention, SDG 4, developing countries

## Abstract

The United Nations' Sustainable Development Goals (SDGs) explicitly acknowledge inclusive and equitable quality education as the primary goal of any global initiatives for early childhood development for children under 5 years with developmental delays and disabilities. Primary education provides the foundation for lifelong learning, vocational attainment, and economically independent living. Globally, the majority (over 90%) of children with developmental disabilities reside in low- and middle-income countries (LMICs). These children are significantly less likely to have foundational reading and numeracy skills, more likely to have never attended school and more likely to be out of primary school, compared to children without disabilities. Concerted and well-coordinated efforts to prepare these children in early childhood for inclusive education constitute a moral and ethical priority for all countries. This paper sets out to examine the concept and dimensions of school readiness for children under 5 years from an extensive narrative review of the literature. It identifies the barriers and challenges for school readiness for children with disabilities and the limitations of the available tools for evaluating school readiness. It concludes by emphasizing the critical role of inter-disciplinary engagement among pediatric caregivers in promoting school readiness in partnership with the families and community where the children reside. Overall, the paper highlights the need for appropriate policy initiatives at the global and national levels to promote school readiness specifically for children under 5 years with developmental disabilities in LMICs, if the aspirational goal of inclusive education by 2030 under the SDGs is to be realized.

## Introduction

Developmental disabilities (or simply “disabilities” hereinafter) are chronic physical, cognitive, speech or language, psychological, or self-care conditions that typically originate during childhood; are likely to continue indefinitely; and require additional coordinated services, support, or other assistance for an extended duration or during a lifetime (1, 2). These conditions include but not limited to hearing impairment, vision loss, cerebral palsy, epilepsy, intellectual disability, autism spectrum disorder, attention-deficit/hyperactivity disorder, speech and language disorders, and specific learning disabilities. Globally, more than 50 million children aged under-5 years are estimated to have disabilities (3). A recent report from UNICEF suggests that, compared to children without disabilities, children with disabilities are 42% less likely to have foundational reading and numeracy skills, 49% more likely to have never attended school, 47% more likely to be out of primary school, 33% more likely to be out of lower-secondary school, 27% more likely to be out of upper-secondary school, and 20% less likely to have expectations of a better life (4). The United Nation's Sustainable Development Goals (SDGs) have provided the political and policy framework for ensuring that children under-5 years with disabilities are promptly identified and supported to benefit from inclusive and equitable quality education (5). SDG 4.2 specifically calls for actions to ensure that all girls and boys have access to quality early childhood development (ECD), care and pre-primary education so that they are ready for primary education by 2030. Thus, school readiness is a critical component of the global health agenda for children under 5 years with disabilities. This has been reinforced by the 2015 Incheon Declaration and Framework for Action for the implementation of Sustainable Development Goal 4 (Education 2030) led by UNESCO (6). It is also consistent with the United Nations Convention on the Rights of the Child (7), and the United Nations Convention of the Rights of Persons with Disabilities (8).

In this mini-review, we set out to: (i) examine the concept and dimensions of school readiness with respect to inclusive education among children under 5 years with disabilities; (ii) identify the barriers and challenges for school readiness for children with disabilities from the perspective of child, school and family/community; (iii) examine the limitations of the available tools for the evaluation of school readiness; and (iv) highlight the role of pediatric caregivers in facilitating school readiness for children with disabilities in low- and middle-income countries (LMICs). The articles and reports used in this narrative review were identified through targeted searches of the PubMed, Scopus and Google using the terms “school readiness” and “childhood disability.” Additional articles were identified from the references of selected publications and reports.

## The concept and dimensions of school readiness

School readiness is a measure of the preparedness of a child, with age-appropriate physical and emotional wellbeing as well as social, language and cognitive or intellectual competencies to succeed in school. The concept of preparedness and competencies for school readiness has evolved with time from a maturational construct (wherein the maturity level of the child was solely responsible for the attainment of appropriate skills helpful for success in school) (9), to a social construct (wherein the child has an active role in becoming ready for school through a wide range of interactions between the child and his environment) (10).

**Figure 1:**
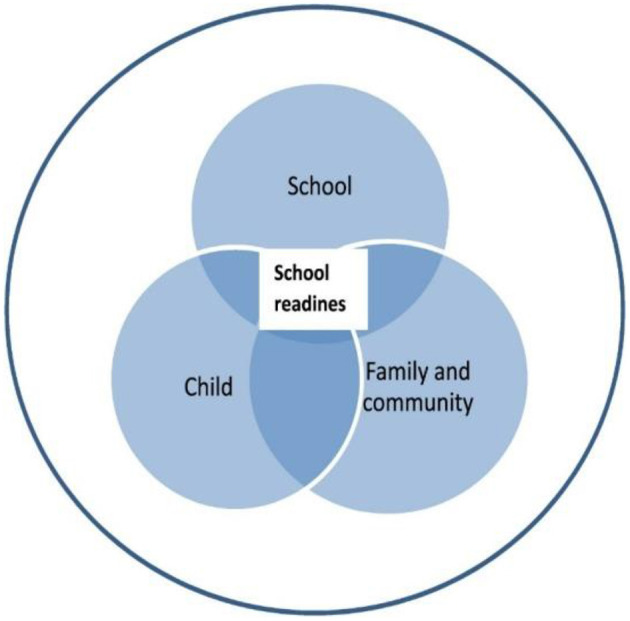
UNICEF's model for school readiness (12).

School readiness comprises three interconnected dimensions: the readiness of the individual child for primary school enrolment and participation; the school's readiness to provide optimal learning environment for the child; and family and community supports that contribute to child readiness for school, as depicted in Figure 1 (11, 12). “Ready children” have skills, abilities and attitudes that are required for a smooth and successful transition to school, such as, self-regulation, early literacy, early numeracy, motor, social-emotional, and executive function skills. “Ready schools” have appropriately trained teachers and high quality of support services to provide smooth transitions for children irrespective of their abilities and at their own pace. Family and community readiness involves parenting beliefs, attitudes, and practices, which are quite varied across cultures and socio-economic groups, as well as community support. These dimensions are applicable to all children. However, children with disabilities have peculiar challenges that require special attention over and above those without disabilities in order to foster school readiness for inclusive education.

## School readiness for children with disabilities

In line with SDG 4.2, school readiness for children with disabilities must be geared toward inclusive education that allows full and effective participation, accessibility, attendance, and achievement along with children without disabilities (6–8). An overview of the three dimensions of school readiness for children with disabilities is presented below.

### Child's readiness for school

The domains of school readiness for any child typically include (i) Health and Physical Development, ii) Emotional WellBeing and Social Competence, (iii) Approaches to Learning, iv) Communicative Skills, and v) Cognition and General Knowledge (13). When children's physical health forms the basis for the development of school readiness skills and successful transition to school (14), the school readiness skills of children with disabilities assume greater importance as they are less likely to engage in the process of education itself. Compared to other children, those with disabilities are less likely to start school, have lower levels of attendance, have lesser chance for higher education, and have lower school retention rates (15–17).

Lack of access to timely detection and intervention services is perhaps the greatest barrier to school readiness faced by children with disabilities (18). Routine newborn screening and developmental monitoring are generally not offered in many LMICs. Where services exist, poverty, discrimination, stigma, and abuse may constitute additional barriers (19). As a result, these children falter in all the essential domains of child development for school readiness (13). Specific disabilities are also associated with unique challenges. For example, children with Autism Spectrum Disorders may experience less emotional readiness as they have more externalizing behaviors and difficulties with self-regulation which adversely affects their engagement in the classroom activities as well as social interactions with teachers and peers (20).

Studies also show considerable impairment in cognition and general knowledge, lower academic scores, increased grade retention and dropout rates among children with ADHD (21). This is because hyperactivity and impulsivity affect social interactions and the so-called normal classroom behaviors like paying attention to the teacher or activities, being able to sit still in the class etc., and interpersonal issues due to poor emotional control.

Preschool age children with Cerebral Palsy have been found to perform well below their peers in areas of mobility, self-care, social interactions, and communication skills. Hence, the need for timely screening and intervention for these children so as to prepare them for school entry (22). Similarly, studies show that enrolment in early intervention services for deaf or hard-of-hearing children well before 6 months of age help establish healthy trajectories of early childhood development, thereby reducing later academic challenges (23).

### School's readiness for the child

For schools to be ready to provide developmentally appropriate education for children with disabilities in an inclusive educational setting, they need to satisfy diverse learning needs and preferences in the present-day classrooms. As one of the goals of inclusive education is not only to accept children with disabilities, but also to welcome them, schools need to bring about systematic changes not only in the way schools' function, but also in the attitudes, beliefs, and value systems of all stakeholders of the school including families and community at large. Studies have shown that although children with disabilities liked attending school most of the time they are discouraged by discrimination, prejudice and non-acceptance from peers (24). Those in school are all too often excluded within the school setting and are not placed with peers in their own age group and receive poor-quality learning opportunities. Hence, the need for proper guidelines for implementing inclusive education in schools (25).

Studies conducted in LMICs have shown that teachers do not have adequate knowledge about disabilities and inclusive education and only few teachers receive requisite hands-on training beyond lectures. As a result, many teachers lack confidence in teaching children with disabilities resulting in the belief that children with disabilities should be taught out of mainstream education system (19). Many schools also lack infrastructural facilities to meet the needs of children with different forms of disability. Schools, therefore, need to be adequately funded and equipped to receive children with disabilities. UNICEF's Child Friendly Schools (CFS) can be considered as a model of school's readiness for children with disabilities (26). The characteristics of child friendly schools are: (i) child-centered approach in teaching and learning; (ii) hygienic; (iii) healthy; (iv) safe-adherence to safety regulations in construction of buildings and playgrounds; (v) protective; (vi) gender sensitive and (vii) inclusive. CFS also links the three dimensions of school readiness by involving the family and community in children's learning and development (26).

### Family/community's readiness for school

Parents play a crucial and indispensable role in fostering school readiness of children with disabilities. Parents act as decision makers on behalf of their children and assist others in making decisions about their children in school related matters. They act as teachers not only at home but also as partners in the classroom. And their role as an advocate for their child also makes them the most important group in the school community setup (25).

However, parents in LMICs must overcome several personal and societal challenges in getting their children with disabilities ready for school. Studies have shown that the main obstacles to transition to primary school for children with disabilities in sub-Saharan Africa are related to stigma, financial limitations including costs to the family, resources in school and travel (19, 24, 27). Problems associated with accessing health care and education facilities also affects children's health, development, and education as these programs and services may often be costly, not inclusive and situated in urban areas. Although some countries have a good network of community-based services for children, there is a dearth of knowledgeable and skilled service providers for disability. Challenges in physically reaching the schools is also a factor affecting schooling for children with disabilities in some communities. Children with disabilities have also been found to miss out on essential vaccinations and basic treatment for common childhood illnesses which compromises their school readiness and smooth transition to formal education (28). Parental empowerment and community enlightenment are needed to foster school readiness for children with disabilities. Parental perception on disabilities, their concerns about school, their perception of benefit from schooling to their child with a disability must also be considered and addressed as appropriate.

## Evaluation of school readiness

Even after almost 50 years of research, the concept of School Readiness and its assessment is an area wherein a consensus among the stakeholders is still emerging (29). Evidence in the literature shows varied approaches to the dimensions of school readiness assessment such as the age at which school readiness should be assessed, which is dependent on the transition age to primary school and varies with the education policies of each country (27, 30–33). The types and dimensions of assessment, as well as the reliability and validity of the assessment tools, especially when test scores form the basis for denial of entry or admission to special education are important. Additional considerations include who the assessor should be, the settings and frequency of assessments, cultural sensitivity of assessment tools, communicating school readiness status of children with their parents and using readiness data for other purposes of curriculum planning. However, the appropriateness of school readiness tools for children with disability remain largely untested in LMICs (27). A summary of available tools is presented in Table 1.

**Table 1:** Instruments for assessing child/school and family readiness.

**(a). Instruments for assessing child's readiness**								
**Name of instrument**	**Assessor**	**Functional domains**	**Age group**	**Feasibility**	**Reliability**	**Validity**	**Scoring**	**Experience with total population implementation**
1. The Jamaica school readiness assessment (JSRA)	Teacher	JSRA has three components: The Eleven Question Screen (EQS) an adapted version of ten question screening, the child behavior rating scale and the early learning scales. The functional domains assessed are development, behavior, early literacy skills, and early numeracy skills, approaches to learning	4 years −4 years 11 months	Feasible for classroom settings where teacher completes the questionnaires based on observation. Based on the normative cutoff points decisions about further evaluations are made	The standardized alpha for the approaches to learning (0.81), early literacy (0.89), and early numeracy (0.87) areas indicated strong internal consistency for all three areas. Internal consistency was also examined for the CBRS, and the standardized alpha was 0.86, also indicating strong internal consistency	Original study showed high sensitivity and specificity for original TQS The CBRS has demonstrated strong predictive validity with reading and math achievement in elementary grades and validated in different cultural contexts	Cut off scores for each of the component instruments has been identified for comparison against normative sample	In Jamaica, Bangladesh and Pakistan TQS had relatively poor sensitivity for serious vision and hearing disorders that had not been previously identified and a low positive predictive value of less than 25% for serious disability. Hence positive screen result therefore needs to be followed by a clinical diagnostic evaluation to confirm the presence or absence of disability
2. Early development instrument (EDI)	Teacher/educator	Physical health and wellbeing, social competence, emotional maturity, language and cognitive development, communication skills and general knowledge	4 to 7 years	An easy to administer paper pencil/digital three-point Likert type scale which can be administered with minimal training, requires only 15–20 minutes for Individual child. This instrument is intended to collect individual child's data but results are not interpreted for individual child and not for diagnostic purposes	Internal consistency (alpha) ranged from 0.84 to 0.96. Test-retest reliability coefficients ranged from 0.82 to 0.94. Inter-rater reliability (as measured by correlation of school-teacher and daycare teacher scores, as well as parent-teacher scores) ranged from 0.36–0.80	Validity studies based on Content validity, response processes, internal structure as well as in relation with other variables like social competence, physical health, emotional maturity, language development three years after initial EDI administration as well as academic outcome at the end of first grade demonstrated good validity	Percentile cut-points, and norm-referenced scores (based on national results from Canada) are available for comparison. Children who score in the lowest 10th percentile on one or more domains are categorized as vulnerable	EDI was finalized in 2000 in Ontario. Most provinces continue to implement the EDI on a regular basis. Many countries have implemented the EDI with suitable adaptations to local settings to ensure validity and relevance across settings
3. The international development and early learning assessment (IDELA)	Trained enumerator/community member	Early numeracy, early literacy, social-emotional development, and motor skills	3.5–6 years	Direct individual skill assessments of children are done for all the 22 items on the instrument through direct child interview and observation, which takes ~30 min for each child. Requires minimal set of materials for administering the test	High inter rater reliability was observed in different settings	All domains of development measured by IDELA are predictive of later academic performance in Early primary school, and the domains of Emergent Literacy and Emergent Numeracy are the strongest predictors of Early Grade Reading Assessment and Early Grade Maths Assessment. Internal consistency calculations were performed for both the overall IDELA instrument and four of the subscales for the countries where IDELA has been administered	75% correct scoring is considered as fine mastery and 25% correct scoring is considered as struggling for overall assessment s and for a particular functional domain	IDELA has been used in 45 countries to assess the ECE interventions aimed at achieving SDG 4.2 goals. Further predictive validity studies that investigate whether there are IDELA score ranges associated with better primary school outcomes are needed before performance benchmarks can be established as per the original study
4. Malawi development assessment tool^**^	Trained health worker	Gross motor, fine motor, language, and social skills	0–6 years	Technically sound and suitable for African rural settings. Could be used by with little training and the items are easy to understand as pictorial representations of many items are provided in the tool.	Overall, reliability was excellent (*k* > 0.75) for 99% (134/136) of interobserver immediate reliability this table, for 89% (121/136) interobserver delayed reliability, and 71% (96/136) of intra-observer–delayed 2-wk assessments	Very high sensitivity (97%), and specificity 82%	Age norms for 25, 50, 75, and 90% percent of the children passing each item was determined which acts as normal reference values for each functional domain milestones	Authors have mentioned that limited resource settings can use this scale for initial assessment of children's development as well as outcome measurement tool for interventions
5. Nursery evaluation scale Trivandrum (Abridged version)	Community health worker	Gross motor development, fine motor development, cognitive development, receptive language development and personal social and expressive language development	48 months-72 months	Brief, simple, cost effective and easy to administer screening tool which requires minimal training and less time for administering in community setting. It provides scope for continuous evaluation of children to monitor their progress after offering inbuilt intervention programs for each item.	NEST abridged is a shorter version of NEST Full version. Psychometric studies of NEST full version have been published in the Indian Academy of Pediatrics Textbook Vth Edition^*^	Psychometric studies of NEST full version have been published in the Indian academy of pediatrics textbook	3rd, 50^th^, and 97th normative Percentile age placements for each item is available for comparison	Large population experiences are yet to be documented for NEST abridged version although it is available for NEST Full version
**(b). Instruments for assessing school's readiness**								
1. School assessment tool (reflection matrix)	Members of school community	It includes seven dimensions of family-school partnerships framework: (i) communicating; (ii) connecting learning at home and at school; (iii) building community and identity; (iv) recognizing the role of the family; (v) consultative decision-making; (vi) collaborating beyond the school; and (vii) participating	Not applicable	Contains individual, school and group assessment proformas. easy to administer, the results of individual assessments are collated onto group assessment proforma. These results after discussion with the members about the school's current stage on each dimension is entered into the school profile overview proforma and the differences in rating between groups discussed and action plans formulated	Not available	Not available	Not available	Not available
2. Checklist to assess the accessibility of schools for children with disabilities	Parents, school administrators, school management committee, civil works personnel	Entry/exit, ramps, stairs, corridors, signage, doors, boards, windows, flooring, drinking water units, toilets, playgrounds and emergency preparedness	Not applicable	The checklist outlines access requirements to comply with the diverse needs of all children, including children with disabilities and to use the guidebook to understand as to improve the accessibility by working on areas identified as requiring improvement. This can be used in planning, designing and implementation of school related construction works or for self-assessment, monitoring and maintenance purpose, third party audits, advocacies for improving accessibility to schools etc.	Not available	Not available	Yes or NO response with a remarks column for noting observations and reference column indicating the required section in the guidebook for improving particular design element	Not available
**(c). Instruments for assessing family's readiness**								
1. Family engagement best practices rubric and assessment	Individuals, teacher groups, family groups, student groups or by the whole school community	Communication, strengthening relationships and capacity, connecting learning at home and at school, recognizing the role of the family, shared decision making, collaborating with community and participating	Not applicable	Based on the individual assessment family engagement action plan to be prepared	Not available	Not available	Three stages of, Developing, Building, Sustaining, within each element to represent a continuum of engagement based on YES/NO/ DON'T KNOW responses for each statement	Not available

* MKC Nair, Babu George. Early detection and early intervention therapy for developmental delay. In: A Parthasarathy, PSN Menon, Piyush Gupta, MKC Nair, editors. IAP Textbook of Pediatrics. 4th ed. New Delhi: Jaypee Brothers; 2009.p.1055-1077.

** Gladstone M, Lancaster GA, Umar E, Nyirenda M, Kayira E, van den Broek NR, Smyth RL. The Malawi Developmental Assessment Tool (MDAT): the creation, validation, and reliability of a tool to assess child development in rural African settings. PLoS Med. 2010 May 25;7(5):e1000273. doi: 10.1371/journal.pmed.1000273. PMID: 20520849; PMCID: PMC2876049.

### Evaluating child's readiness

Tools for assessing school readiness in children in general are varied and consists of screening tests, diagnostic tests, and generic school readiness tests. It was observed that only few instruments considered the contextual aspects of children's learning, the quality of environment (34, 35), the individual and group differences in the patterns of child development as well as impairment or disability (31). However, most of the tools conserved the biological-maturational aspect linked to the achievement levels in various domains of development suitable for each age.

For young children (0–6 years), there are five conditions for which routine screening programs have been recommended and implemented in several countries: (i) congenital metabolic conditions, (ii) hearing, (iii) vision, (iv) developmental and behavioral disorders, and (v) autism spectrum disorder (ASD) (36). School Readiness module and scale to assess the outcome of the intervention in pre-schoolers with autism spectrum disorder has been developed and validated in a developing country but is yet to be widely used (37). Some 32.6% of 4-year-olds assessed using The Jamaica School Readiness Assessment (JSRA) in Jamaica in 2017 and 2018 were identified as having at least one developmental problem (36). Early Development Instrument (EDI), a teacher administered tool for assessing the development of children in the age group of 3.5–6.5 years, has been widely used in Canada and is in use in Ethiopia, Malawi, and Mozambique (38). The International Development and Early Learning Assessment (IDELA) is a global tool administered by trained enumerators to assess early learning and development of children in the 3.5–6-year age group (39); but school readiness threshold is not available and certain IDELA score range is not indicative of developmental delay. IDELA has been used in 45 countries and has been adapted for use in Bhutan (31). Malawi Development Assessment tool is another tool with good specificity in identifying developmental delay in children from low-income settings. This has been used in Zimbabwe, Pakistan, Kenya, Uganda, Bangladesh, Tanzania, and Nepal (39).

Lastly, the Nursery Evaluation Scale Trivandrum (Abridged Version) is a simple, cost-effective screening tool to assess the development of children from 48 months to 72 months to be used in the community settings by community health workers (40). The 3rd, 50^th^, and 97th percentile age placement in months have been provided.

### Evaluating school's readiness

School Assessment Tool (Reflection Matrix) has been designed to assist the stakeholders of the school community to assess the current family and community engagement practices and thereby implementing strategies to strengthen them (41). This assessment tool helps schools understand their position on the continuum of engagement and where further development is required. This tool aligns with the seven dimensions of Family-School Partnerships Framework: (i) communicating; (ii) connecting learning at home and at school; (iii) building community and identity; (iv) recognizing the role of the family; (v) consultative decision-making; (vi) collaborating beyond the school; and (vii) participating (41, 42). This tool can be culturally adapted for LMICs because of its simplicity.

Government of India launched Accessible India Campaign (Sugamya Bharat Abhiyan) in 2015 to achieve universal accessibility for persons with disabilities. A checklist was developed to assess the accessibility of schools in India for children with disabilities as part of the guidebook titled: “Making Schools Accessible to Children with Disabilities” (43).

### Evaluating family's readiness

Specific tools aimed at assessing family's readiness for school are rare, even in high-income countries. A tool currently used in Australia under the Albuquerque Public Schools Family and Community Engagement Policy, addresses issues that may be considered in evaluating parent engagement in school readiness (44).

## Intervention programs for school readiness

Evidence shows that disadvantaged students with or at risk of disabilities are those making the most dramatic gains from ECD programs and in turn from school readiness programs (45). Examples of intervention programs to facilitate school readiness in children with disabilities include the “Head Start Program” in the USA (46), and the Integrated Child Development Services (ICDS) in India (47).

### Head Start Programs (USA)

The “Head Start” and “Early Head Start” Programs were launched in 1965 targeted at children from birth to 5 years of age hailing from low-income families, and foster care systems. The services are offered at no charge to parents. Children with disabilities and special needs are also catered for in the Head Start Programs. The Early Head Start component caters to the needs of expectant mothers, infants, and toddlers and are mostly provided in the child's own home through weekly home visits, while the Head Start Program is aimed at promoting school readiness for all children 3–5 years of age through center-based activities (46). The Head Start Program is highly successful and exemplifies a useful framework for developing culturally appropriate intervention programs in LMICs.

### Integrated child development services (ICDS)—India

Integrated Child Development Services launched in 1975, is one of the world's largest and unique ECD programs (47). The objectives of this program are: (i) to improve the health and nutritional status of children under 6 years; (ii) to lay the foundation for the physical, psychological, and social development of the child; (iii) to reduce malnutrition, mortality, morbidity as well as school dropout rates; (iv) to promote inter department coordination at the policy as well as implementation level so as to promote child development; and (v) to enhance mother's capability to meet the health and nutritional requirements of their children through proper health and nutrition education. ICDS focusses on an integrated and life cycle approach in delivering services to its beneficiaries: children under 6 years of age, pregnant women, and lactating mothers. All the services of ICDS are provided through its grassroot level center called the Anganwadi center, manned by Anganwadi worker and an assistant. The services provided to children under 6 years of age, adolescent girls, and pregnant and lactating mothers through Anganwadi are: supplementary nutrition (to bridge the gap between the Recommended Dietary Allowances (RDA) and the Average Daily Intake (ADI) of the target group), health check-up, referral services and immunization. ICDS also aims at breaking the vicious cycles of malnutrition, mortality and morbidity and reduced learning capacity as well as provide non formal education to children between 3 to 6 years of age (47).

Anganwadi workers have been trained in identifying developmental delay in children from birth to 2 years of age using Trivandrum Developmental Scale developed at Child Development Center, Trivandrum and to assess school readiness as a continuous assessment program using Nursery Evaluation Scale Trivandrum in 2- to 6-year-old children. Anganwadi workers are also trained in providing family Life education sessions to adolescents belonging to their Anganwadi area. In the financial year 2021, more than 89 million mothers and children had benefited from ICDS (48). One evaluation study conducted in three states in India demonstrated that ICDS also has a significant benefit for the mental development of the children (49).

## Role of pediatric caregivers in promoting school readiness

The scientific, ethical, and political framework for optimizing school readiness for inclusive education for children with disabilities as envisaged by the SDGs has been reported in the literature (18, 50, 51). Pediatric caregivers, including nurses, physicians and other primary care professionals, community health workers and rehabilitation specialists have a significant role in promoting school readiness for all children, right from birth through pediatric consultations as well as advocacy (52, 53). Available evidence from both pediatrics and education shows that children with disabilities start school farther behind than their peers without disabilities (4). Inter-disciplinary work between pediatrics and education to drive the implementation of evidence-based solutions will ultimately improve the developmental trajectory for better outcomes for these children. For instance, the healthcare system is the only sector that enjoys highest contacts with children before school entry, particularly, through routine immunization programs in communities with high rates of births outside hospitals. National guidelines similar to the policy document from the American Academy of Pediatrics (AAP) on early detection and intervention provide caregivers with opportunities for improving physical, socio-emotional and educational health of young children with other advocacy groups (53). Ensuring children's regular and timely visits to the well-child clinics is a way of ensuring healthy child development and school readiness. These visits, apart from screening for risks factors and the early identification and intervention for disabilities, provide opportunities for pediatric caregivers to monitor and ensure parental education on children's growth, development, and nutrition, handling behavioral issues, as well as the importance of quality parent-child interaction within a positive home environment. The importance of family-centered services cannot be over-emphasized (54–56).

Community support systems through home visits can be used for promoting school readiness, family support programs and early intervention services (57). Kindergarten screening, rather than a gatekeeping test for age-eligible children to enter school should be a tool to guide planning, curriculum, and instruction to support developmental and academic achievement for diverse groups of children. A school readiness curriculum for increasing the pediatric resident's knowledge and confidence in addressing school readiness in clinics has also been developed and evaluated for pediatric residents (58). The International Pediatric Association has also issued a position statement that addresses the training needs of the pediatric service providers (59). These recommendations can be adapted for use in LMICs within the pediatric community of caregivers to ensure that efforts to facilitate early detection and intervention for children with disabilities are appropriately geared toward school readiness.

## Conclusion

Inclusive education has been acknowledged as a global priority for children with disabilities under the SDGs. However, there is limited evidence of progress toward systematic promotion of school readiness in LMICs across the dimensions of child readiness, school readiness and family/community readiness. Intervention programs in early childhood for children with disabilities are still not explicitly structured and evaluated to facilitate school readiness for inclusive education. Policy interventions to address barriers to school readiness for inclusive education among families, the community, and schools at the country level in LMICs should be considered. Additionally, there is an urgent need to train and empower all pediatric health caregivers to recognize and embrace school readiness for children with disabilities as an early childhood development priority as envisioned by the SDGs framework for global child health, inclusive education, and development.

## Author contributions

MN and RR conceptualized and drafted the manuscript. BO critically reviewed the draft and suggested essential edits. All authors contributed to revising the manuscript, and approved the final version as submitted.
